# Direct-Acting Antivirals and the Risk of Hepatitis B Reactivation in Hepatitis B and C Co-Infected Patients: A Systematic Review and Meta-Analysis

**DOI:** 10.3390/jpm12121957

**Published:** 2022-11-26

**Authors:** Joo Hyun Oh, Dong Ah Park, Min Jung Ko, Jeong-Ju Yoo, Sun Young Yim, Ji-Hyun Ahn, Dae Won Jun, Sang Bong Ahn

**Affiliations:** 1 Department of Internal Medicine, Nowon Eulji Medical Center, Eulji University School of Medicine, Seoul 01830, Republic of Korea; 2 Division of Healthcare Technology Assessment Research, National Evidence-based Healthcare Collaborating Agency (NECA), Seoul 04933, Republic of Korea; 3 Department of Internal Medicine, Soonchunhyang University Bucheon Hospital, Bucheon 14584, Republic of Korea; 4 Department of Internal Medicine, Korea University Hospital, Seoul 02841, Republic of Korea; 5 Department of Internal Medicine, Hanyang University College of Medicine, Guri 11923, Republic of Korea; 6 Department of Internal Medicine, Hanyang University College of Medicine, Seoul 04763, Republic of Korea

**Keywords:** hepatitis B virus, hepatitis C virus, direct–acting antivirals, reactivation

## Abstract

Hepatitis B (HBV) reactivation was observed to be more than 10% in patients receiving interferon-based therapy for hepatitis C (HCV) co-infection. At present, when direct-acting antiviral (DAA) has become the main treatment for HCV, there are few large-scale studies on the reactivation of HBV in these population. We studied HBV reactivation risk and prophylactic HBV treatment efficacy in HBV/HCV co-infected patients receiving DAA therapy. Relevant studies were selected from the Ovid-Medline, Ovid-EMBASE, Cochrane Central Register of Controlled Trials, KoreaMed, KMbase, and RISS databases through 4 September 2020. Data pooling was carried out using the random-effects method. We identified 39 articles with 119,484 patients with chronic (*n* = 1673) or resolved (*n* = 13,497) HBV infection under DAA therapy. When the studies were pooled, the HBV reactivation rate was 12% (95% confidence interval (CI) 6–19, I2 = 87%), indicating that this population needs careful attention. When stratified by baseline HBV DNA, the undetectable HBV DNA group showed a significantly lower risk of reactivation than the detectable HBV DNA group (odds ratio (OR) 0.30, 95% CI 0.11–0.86, I2 = 0%). Prophylactic HBV therapy reduced HBV reactivation risk (OR 0.25, 95% CI 0.07–0.92, I2 = 0%). Patients with a resolved HBV infection showed a negligible rate (0.4%) of HBV reactivation. In conclusion, patients with detectable HBV DNA levels warrant careful monitoring for HBV reactivation and may benefit from preventive anti-HBV treatment.

## 1. Introduction

Hepatitis B virus (HBV) or hepatitis C virus (HCV) infection is the leading cause of chronic liver disease worldwide. Since the two viruses have common transmission pathways, from 1.5% to 14% patients living in endemic areas can be infected with both HBV and HCV [1]. Patients with HBV/HCV co-infection have more severe inflammation and fibrosis and a higher risk of cirrhosis than those with a mono-infection [2]. Furthermore, HBV and HCV co-infection synergistically affect the development of HCC [3]. Patients with HBV or HCV should, thus, be evaluated for co-infection and treated appropriately if co-infection is detected [4,5]. As HCV is usually dominant over HBV, co-infected patients are often treated like those with HCV mono-infection [6].

Previously, interferon-based therapy was the standard of care for HCV patients. Interferon also shown favorable results in HBV patients, with 5–10% of interferon-treated patients achieving HBsAg seroconversion [7]. However, HBV/HCV co-infected patients had undesirable results that HBV-infected patients, with from 19.1% to 36.4% of patients developing HBV reactivation following interferon-based treatment [8]. This phenomenon might be because treatment with HCV alters the activity of HBV. At present, with a better understanding of the HCV genome and proteins, direct-acting antiviral (DAA) therapies have been developed, resulting in significant improvements in the effectiveness, safety, and tolerability of HCV therapies. Unfortunately, there are increasing concerns regarding HBV reactivation following DAAs therapy among patients with HCV and concurrent HBV infection. According to the US Food and Drug Administration, 2 out of 24 HBV/HCV co-infected patients treated with DAAs died, and 1 required liver transplantation due to HBV reactivation between November 2013 and July 2016 [9]. Recently, DAA-induced HBV reactivation has been reported in several studies. Chen et al. described that the risk of HBV reactivation in co-infected patients was estimated at 12.2% [10]. Another recent randomized trial showed that HBV reactivation occurred in 50% of patients, and entecavir prophylaxis significantly lowered the rate of reactivation [11].

Since reactivation rates vary across studies, a comprehensive review and meta-analysis are required. In addition, it is unclear which factors increase the chance of reactivation and the need for pre-emptive antiviral therapy. To address this issue, we performed a meta-analysis to estimate the risk of HBV reactivation in patients with HBV/HCV co-infection receiving DAA treatment.

## 2. Materials and Methods

### 2.1. Data Search Strategy

We searched Ovid-medline, Ovid-EMBASE, Cochrane Central Register of Controlled Trials (CENTRAL), KoreaMed, KMbase, and RISS databases from their inception to September 2020 using the following search terms and their variations: hepatitis B virus, AND hepatitis C virus, DAA, and HBV reactivation (Supplementary Table S1). The data search was limited to English- or Korean-language articlesThe protocol for this review was registered in advance in the PROSPERO register (International Prospective Register of Systematic Reviews (CRD42021241245). The review followed the Preferred Reporting Items for Systematic Reviews and Meta-Analyses (PRISMA) guidelines and the Meta-analysis of Observational Studies in Epidemiology (MOOSE) checklist.

### 2.2. Study Selection

Patients were eligible if they had both chronic HCV infection treated with DAAs and active (HBsAg (+)) or resolved (HBsAg (−) but HBcAb (+)) HBV infection. Studies were included if they met the following inclusion criteria: (1) studies evaluated in patients with HBV/HCV co-infection, (2) studies using DAA, and (3) studies reporting HBV reactivation. Articles were excluded for the following reasons: (1) non-human or pre-clinical studies; (2) reviews or meta-analyses, guidelines, consensus, comments, or letters; (3) non-Korean or non-English language; and (4) absence of detailed data. Since the first DAAs were approved by the FDA in 2011, we also excluded studies published before 2010 [9].

The primary endpoint was HBV reactivation, defined as: (1) reappearance of HBV DNA, and (2) more than one or two log elevations of serum HBV DNA from the baseline level [7,12,13].

### 2.3. Data Extraction and Quality Assessment

Data extraction and quality assessment were conducted by two authors (JJY and SBA), independently. Full-text materials were examined independently following the screening of titles and abstracts. Discrepancies between authors were resolved by consensus or consultation with a third author (DAP or DWJ). To assess the risk of bias, we utilized the Risk of Bias Assessment tool for Non-randomized Studies (RoBANS) tool (Cochrane Bias Method group, version 2.0). Further information on the outcome of the risk of bias is presented in the Supplementary Materials.

### 2.4. Statistical Analysis

The meta-analysis was performed using a random-effects model or fixed-effects model and presented pooled odds ratios (OR) and 95% confidence intervals (CI). Heterogeneity was visually checked using a forest plot and determined based on the Cochrane Q statistic (*p* < 0.10) and *I*^2^ statistics (≥50%). Subgroup analysis was used to explain the heterogeneity among the studies. Subgroup analysis was conducted by study area, baseline HBV DNA level, and HBV prophylaxis. We also estimated the pooled OR for HBV reactivation in resolved HBV infected patients. If possible (more than ten articles included in the analysis), a method using funnel plot and Egger’s test or AS-Thompsom’s test was performed to identify publication bias. The meta-analysis was performed using the RevMan software (version 5.3, Cochrane library) and R statistical software (version 4.1.1, The R Foundation for Statistical Computing, Vienna, Austria). Statistical significance was considered at *p* < 0.05.

## 3. Results

### 3.1. Baseline Characteristics of Included Studies

**Figure 1:**
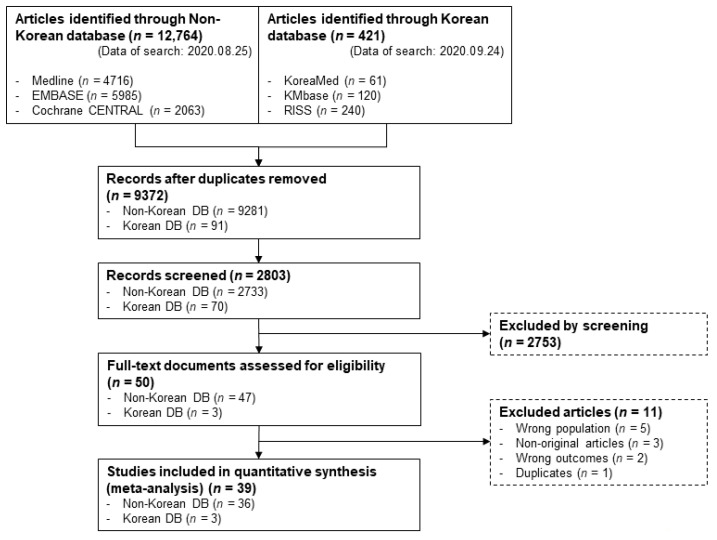
Literature search process for the systematic review and meta-analysis.

This systematic review identified a total of 13,185 records (Figure 1). After eliminating duplicates, 2803 records were screened for titles and abstracts, and 50 articles from the reference lists were selected for full-text review. Finally, 39 studies were included in the quantitative analysis. These studies included 1673 patients with HBV/HCV co-infection in 35 articles and 13,497 resolved HBV (HBsAg-negative, anti-HBc-positive) patients in 27 studies (Table 1). Of the 39 studies, 12 (30.7%) studies were retrospective cohorts, and 26 (66.6%) studies were prospective studies from Europe (Italy, Germany, Spain, Poland, Portugal), Asia (Japan, China, Taiwan, Korea, Pakistan), and North America (United States). All included articles were published between 2015 and 2020.

### 3.2. DAA-Associated HBV Reactivation in HBsAg (+) Patients

**Figure 2:**
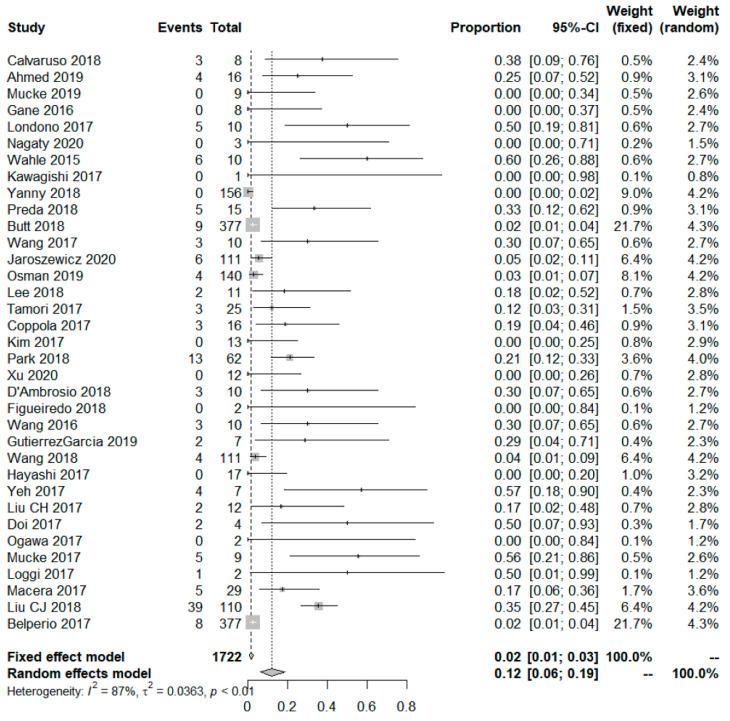
Overall risk of HBV reactivation.

In the 35 studies that reported data on HBsAg-positive patients receiving DAA therapy, the pooled HBV reactivation rate was 12% (95% confidence interval (CI) 6–19). Significant heterogeneity was observed among the studies (I^2^ = 87%). A forest plot of the meta-analysis is shown in Figure 2.

### 3.3. Subgroup Analysis

Subgroup analyses were performed according to the study location (east or west), baseline HBV DNA levels, and administration of prophylactic anti-HBV treatment. There were 35 studies that identified the origin of the research, 15 studies from the east, and 20 from the west. The pooled HBV reactivation rate was significant in both areas, and eastern studies showed a higher reactivation rate (17%) than Western studies (11%) (Supplementary Figure S1).

**Figure 3:**
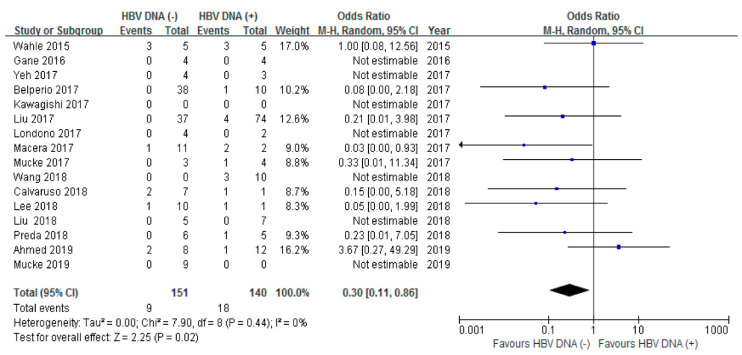
The risk of HBV reactivation according to baseline HBV DNA.

The risk of HBV reactivation was further analyzed in 16 studies that reported the baseline HBV DNA levels. Undetectable HBV DNA was defined as an HBV DNA level < 20 IU/mL. Patients with undetectable HBV DNA showed a decreased risk of reactivation than those with detectable HBV DNA (≥20 IU/mL) (OR 0.27, 95% CI 0.07–1.01, I^2^ = 0%) (Figure 3).

**Figure 4:**
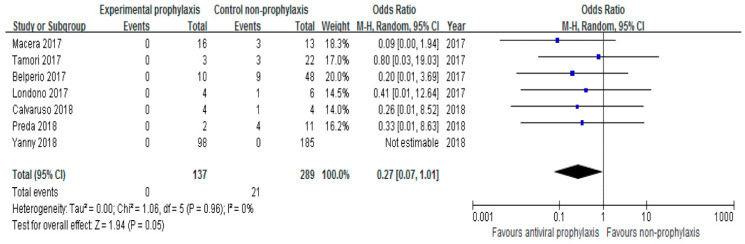
The risk of HBV reactivation according to prophylactic anti-HBV therapy.

The risk of HBV reactivation was compared between patients who received prophylactic anti-HBV therapy and those who did not in seven studies. Although the pooled risk was lower in patients receiving prophylactic HBV therapy, statistical significance was not reached (OR 0.27, 95% CI 0.07–1.01, I^2^ = 0%) (Figure 4). There was no evidence of heterogeneity (I^2^ = 0%, *p* = 0.96). Due to the homogeneity of the studies, a fixed-effects model was employed, and the pooled risk was significantly lower for those who received prophylactic HBV therapy (OR 0.25, 95% CI 0.07–0.92).

### 3.4. DAA-Associated HBV Reactivation in Resolved HBV Patients

Most pooled studies of HBsAg-negative and HBcAb-positive patients receiving DAAs reported that there was no HBV reactivation. However, 5 of the 27 articles demonstrated reactivation. In the meta-analysis, the pooled reactivation risk was 0.4% in patients with resolved HBV (95% CI 0.002–0.01, I^2^ = 66.6%) 

### 3.5. Risk of Bias

In the Supplementary Figures S1–S3, the risk of bias is shown. Excepting participant selection, the majority of categories exhibited a minimal risk of bias (Supplementary Figure S2).

## 4. Discussion

The present meta-analysis demonstrated a significant incidence of HBV reactivation in patients with HCV/HBV co-infection who were treated with DAA (12%). The risk factors for HBV reactivation were detectable baseline HBV DNA levels and the absence of HBV prophylaxis, indicating that HBV suppression prior to DAA therapy may prevent HBV reactivation. In contrast, the probability of HBV reactivation was minimal (0.4%) in patients with a resolved HBV infection.

Several systematic reviews have evaluated the risk of HBV reactivation in patients with HBV/HCV co-infection receiving antiviral HCV treatment [10,52,53,54]. The reported rate of HBV reactivation was 12.2% to 24.0% in patients with HBsAg positivity, and 0.1% to 1.4% in those with resolved HBV infection. Although the findings were in line with those of our study, these studies were limited by the sample size and type of HCV therapy. The study population was relatively small (n = 148–425), and two of the four systematic reviews only focused on patients receiving interferon (IFN)-based treatment rather than DAA therapy. Furthermore, subgroup analysis according to baseline HBV DNA level or the administration of pre-emptive antiviral therapy was not performed. In the present study, we thoroughly reviewed the literature and included studies using DAA rather than IFN-based treatment based on the inclusion criteria. We included a sufficient number of studies (35 studies with HBsAg-positive patients (n = 1673) and 27 studies with resolved HBV patients (n = 13,497)) published between 2015 and 2020 and performed a detailed subgroup analysis. Based on a large study population and recently published articles, we found that the risk of HBV reactivation was significant, especially in patients with detectable HBV DNA levels. Patients in these circumstances should be closely monitored, and preventive antiviral HBV therapy may be beneficial. Additionally, more attention is required for HBV reactivation when employing DAA therapy in the eastern region.

It is well known that the suppressed immune system induced by cytotoxic chemotherapy or immunosuppressants accelerates HBV reactivation in patients with a chronic or previous history of HBV infection [13]. Interestingly, the side effects of the DAA therapy were similar. Although this needs to be elucidated, several plausible mechanisms for this exist. First, the direct inhibitory mechanism of HCV was eliminated by DAA. In vitro studies have shown that HCV core protein (NS2 and NS5a) has an inhibitory effect on HBV replication [55,56] by suppressing HBV enhancer 1 [34] or the HBx protein [57]. In addition, it has been suggested that these two viruses compete for a limited replicative space [58]. Second, innate and/or adaptive immune responses may explain the indirect mechanisms [4]. HCV infection activates the host immune system. Pattern recognition receptors, including retinoic acid-inducible gene I and toll-like receptor 3, activate various immune response pathways, such as IFN and NF-kB [59], resulting in cytokine production and the subsequent overexpression of antiviral IFN-stimulated genes [60]. HCV may evade immune responses by mutation or signal transduction disruption, but these responses may suppress HBV. Therefore, treating HCV infection would lead to the loss of HBV suppression, which might result in reactivation.

Whether HBV reactivation is associated with baseline HBV DNA levels before DAA treatment is still controversial. In this study, we discovered that patients with detectable HBV DNA levels had a greater risk of reactivation than those with lower HBV DNA levels. In contrast, two systematic reviews showed that reactivation risk was similar between patients with undetectable HBV DNA and those with detectable HBV DNA. However, these studies reported that HBV reactivation-related hepatitis was significantly lower in patients with undetectable HBV DNA. Therefore, HBV DNA levels should be monitored before and during the DAA therapy.

Pre-emptive nucleos(t) ide (NUCs) treatment is recommended in patients with moderate (1–10%) to high-risk (≥10%) HBV reactivation undergoing cytotoxic chemotherapy or rituximab [12]. In the present study, the preventive effect of prophylactic NUCs was insignificant when the random-effects model was used. However, since the heterogeneity across studies was minimal, the fixed-effects model could be used for analysis. We discovered that preemptive HBV therapy significantly inhibited HBV reactivation. This discrepancy might be due to the small number of studies that were assessed (seven studies) and the fact that various NUCs (low- and high-genetic barrier NUCs) were analyzed rather than a single NUC. Since prophylactic NUCs therapy lowers mortality in patients with HBV reactivation, we believe that patients with detectable HBV DNA or impaired liver function prone to hepatitis require empirical antiviral therapy. Further studies are required to clarify these hypotheses.

The rate of HBV reactivation was negligible in patients with previous HBV infection (0.4%). Several studies have identified the risk factors associated with reactivation in these patients. Guo et al. reported that elevated liver enzyme levels might be associated with a risk of HBV reactivation in patients with resolved HBV [61]. On the other hand, retrospective and prospective studies have demonstrated that negative (<10 mIU/mL) or low anti-HBsAb (<100 mIU/mL) at baseline is a significant predictive factor for HBV reactivation [62,63,64]. Although the incidence of HBV reactivation is low, HBV DNA levels should be monitored throughout DAA therapy in resolved HBV patients, especially in those with risk factors.

This study has several limitations. First, there was significant heterogeneity among the analyzed studies. Various DAA regimens and HCV genotypes were the main sources of heterogeneity. Second, the definition of HBV reactivation varied among studies. We allowed for individual definitions of HBV reactivation because the definition varied among societies and changed over time. For instance, the 2015 American Gastroenterological Association guidelines defined HBV reactivation as de novo detectable HBV DNA if the baseline level was undetectable or a 10-fold increase in HBV DNA levels if the baseline level was detectable [13]. The European Association for the Study of the Liver guidelines published in 2016 defined an increase in viral load to >2000 IU/mL in conjunction with an increase in ALT [7]. However, according to the 2018 American Association of the Study of Liver Disease guidelines, HBV reactivation in HBsAg-positive patients is characterized as one of the following: (1) more than 2-log (100-fold) increase in HBV DNA over the baseline; (2) HBV DNA more than 3-log (1000) IU/mL in a patient with previously undetectable HBV DNA; (3) HBV DNA more than 4-log (10,000) IU/mL if the baseline level is unavailable [12]. Since the analyzed studies were published between 2015 and 2020, we chose a liberal definition of a 1–2-log increase in HBV DNA. Finally, further studies are warranted to elucidate the clinical utility, safety, and cost-effectiveness of prophylactic HBV antiviral prophylaxis. 

## 5. Conclusions

The present study demonstrated a significant risk of HBV reactivation in patients with HBV/HCV co-infection undergoing DAA treatment. This meta-analysis indicated that co-infected patients should be closely monitored for the development of HBV reactivation and be considered to initiate HBV prevention before initiating DAAs.

## Figures and Tables

**Table 1:** Characteristics of studies in the final analysis (*n* = 39).

Author, Year	Country	Study Design	Population	HBsAg(+) Population	HBsAg(−), Anti-HBc(+) Population	Genotype	DAA
Wahle 2015 [14]	Brazil	Retrospective	10	10	0	NA	DAA
Gane 2016 [15]	USA	Prospective	8	8	NA	1	LDV/SOF
Wang 2016 [16]	China	Prospective	355	10		1,2,3,6	SOF + DCV/LDV + SOF/OMB + PTR + DAS
Sulkowski 2016 [17]	Taiwan/Korea	Prospective	178	0	103	NA	LDV + SOF
Kim 2017 [18]	Korea	Prospective	821	13	NA	NA	DAA
Londono 2017 [19]	Spain	Prospective	352	10	64	1,2,3,4	OBV/PTV ± DSV ± RBV LDV/SOF ± RBV DCV/SOF ± RBV SOF + RBV DCV/SMV
Kawagishi 2017 [20]	Japan	Retrospective	85	1	82	1,2	LDV/SOF DCV/ASV SOF + RBV
Wang 2017 [21]	China	Prospective	327	10	124	1,2	LDV/SOF, DCV/SOF, OBV/PTV + DSV
Tamori 2017 [22]	Japan	Prospective	160	25	765	1,2	LDV/SOF DCV/ASV OMB/PTR SOF + RBV
Coppola 2017 [23]	Italy	Prospective	16	16	0	NA	DAA
Ancha 2017 [24]	USA	Retrospective	941	0	188	NA	DAA
Hayashi 2017 [25]	Japan	Retrospective	256	17	80	NA	DAA
Yeh 2017 [26]	Taiwan	Prospective	64	7	57	NA	LDV/SOF ± RBV, SOF + RBV, OMB/PrOD
Liu 2017 [27]	Taiwan	Prospective	93	12	81	1,2	LDV/SOF, LDV/SOF + RBV, SOF + RBV, PrOD, PrOD+RBV
Doi 2017 [28]	Japan	Prospective	461	4	75	1,2	DAA
Ogawa 2017 [29]	Japan	Retrospective	183	2	63	1,2	LDV, SOF
Mucke 2017 [30]	Germany	Prospective	848	9	272	1,2,3,4	LDV, SOF RBV, OBV/PTV + DSV RBV, DCV, SOF + RBV
Loggi 2017 [31]	Italy	Prospective	137	2	42	1,2,3,4	SOF ± RBV, SOF + LDV ± RBV, SOF + SMV ± RBV, PrOD ± RBV
Macera 2017 [32]	Italy	Prospective	29	29	0	NA	DAA
Belperio 2017 [33]	USA	Retrospective	62290	377	7295	NA	DAA
Tang 2017 [34]	Unknown	Unknown	192	0		NA	LDV/SOF, PrOD, SOF/VEL, GZR/EBR
Calvaruso 2018 [35]	Italy	Retrospective	104	8	37	1,2,3,4	LDV/SOF ± RBV DCV/SOF + RBV SMV/SOF + RBV OBV/PTV/DSV + RBV SOF + RBV
Yanny 2018 [36]	USA	Retrospective	283	156	127	1,4	LDV + SOF
Preda 2018 [37]	Romania	Prospective	2070	15	NA	1	OBV/PTV/DSV + RBV
Butt 2018 [38]	USA	Retrospective	DAA:34632, PEG:23475	DAA group:3322, PEG:1751	DAA:9343, PEG:5005	NA	SOF/SMV, SOF/LDV, PrOD, SOF/RBV
Lee 2018 [39]	Taiwan	Retrospective	153	11	53	1,2	SOF + LDV, PrOD, DCV + ASV
Park 2018 [40]	Korea	Retrospective	62	62	NA	NA	IFN: 34, DAA:24
D’Ambrosio 2018 [41]	Italy	Prospective	692	10	301	1	DAA
Figueiredo 2018 [42]	Portugal	Prospective	329	2	123	NA	DAA
Wang 2018 [16]	China	Prospective	111	111	0	1,2	LDV/SOF
Liu 2018 [43]	Taiwan	Prospective	111	110	0	1,2	LDV + SOF
Ahmed 2019 [44]	Egypt	Prospective	20	16	20	NA	SOF + DAC + RBV: SOF + DAC:7, SOF + SIM:5
Mucke 2019 [45]	Germany	Prospective	822	9	263	1,2,3,4	LDV, SOF ± RBV, OBV/PTV + RBV, OBV/PTV+DSV ± RBV, SMV, SOF ± RBV, DCV, SOF ± RBV,SOF + RBV
Osman 2019 [46]	Egypt	Prospective	140	140		NA	SOF + DCV
GutierrezGarcia 2019 [47]	Spain	Prospective	1337	7	356	NA	DAA
Tahir 2019 [48]	Pakistan	Retrospective	260	0	51	NA	DAA
Nagaty 2020 [49]	Egypt	Prospective	287	3	38	NA	SOF + DCV, SOF + DCV + RBV
Jaroszewicz 2020 [50]	Poland	Retrospective	10152	111	1239	1,3,4	OBV/PTV/RBV ± DSV ± RBV, SOF, GZR/EBR ± RBV
Xu 2020 [51]	China	Prospective	113	12	0	1,2,3	SOF/VEL

Abbreviations: NA, not available; RCT, randomized controlled trial; SOF, Sofosbuvir; RBV or r, Ribavirin; SMV, Simeprevir; DCV, Daclatasvir; LDV, Ledipasvir; GLE, Glecaprevir; PIB, Pibrentasvir; EBR, Elbasvir; GZR, Grazoprevir; PrOD, paritaprevir/ritonavir with ombitasvir and dasabuvir; OMB, Ombitasvir; PTR, Paritaprevir; r, ritonavir; DAS, Dasabuvir; ASV, Asunaprevir; VEL, Velpatasvir.

## Data Availability

Clinical trial number: PROSPERO (International Prospective Register of Systematic Reviews), CRD42021241245.

## Supplementary Materials

The following supporting information can be downloaded at: https://www.mdpi.com/article/10.3390/jpm12121957/s1, Figure S1: The risk of HBV reactivation by area, Figure S2: Risk of bias, Figure S3: Funnel plot for analysis publication bias; Table S1: Search strategy and results.
