# Supplementary material for: Direct-Acting Antivirals and the Risk of Hepatitis B Reactivation in Hepatitis B and C Co-Infected Patients: A Systematic Review and Meta-Analysis
Source: J Pers Med. 2022 Nov 26;12(12):1957. doi: 10.3390/jpm12121957 (PMC9781230; doi:10.3390/jpm12121957)
Supplement: Supplementary file 1 [file jpm-12-01957-s001.zip › jpm-1987938-supplementary.pdf]

Supplementary Material

Direct-acting antivirals and the risk of hepatitis B reactivation in hepatitis B and C co-infected patients: A systematic review and meta-analysis

Table of Contents

I. Supplementary Materials and Methods……………………….………………..…..….2

Search strategy and results

II. Supplementary Figures....……………..…………………………...….……………..5

The risk of HBV reactivation by area

Risk of bias

I. Supplementary Materials and Methods

1. Search strategy and results

1-1. Ovid-Medline (Ovid MEDLINE(R) ALL 1946 to August 25, 2020

No.	Keywords	Results
1	exp Hepatitis C, Chronic/	24430
2	chronic adj5 (hepatitis C or hepacivirus* or hcv).mp.	36077
3	1 OR 2	36077
4	exp Antiviral agents/	357256
5	exp Protease Inhibitors/	203080
6	exp Nucleic acid synthesis inhibitors/	162317
7	(direct* acting anti?viral*) or DAA or ((protease or polymerase) adj3 inhibitor*).mp.	69366
8	(ledipasvir OR sofosbuvir OR elbasvir OR grazoprevir OR velpatasvir OR voxilaprevir OR glecaprevir OR pibrentasvir OR ombitasvir OR paritaprevir OR ritonavir OR dasabuvir OR simeprevir OR boceprevir OR telaprevir OR asunaprevir OR daclatasvir OR Harvoni OR Zepatier OR Epclusa OR Vosevi OR Mavyret OR Maviret OR 'Holkira Pak'OR 'Viekira Pak'OR Viekirax OR Exviera OR Technivie OR Galexos OR Sovaldi OR Victrelis OR Incivek OR Incivo OR Olysio OR Sunvepra OR Daklinza).mp.	12541
9	4 or 5 or 6 or 7 or 8	680628
10	3 and 9	18531
11	(Randomized Controlled Trials as Topic/ or randomized controlled trial/ or Random Allocation/ or Double Blind Method/ or Single Blind Method/ or clinical trial/ or clinical trial, phase i.pt. or clinical trial, phase ii.pt. or clinical trial, phase iii.pt. or clinical trial, phase iv.pt. or controlled clinical trial.pt. or randomized controlled trial.pt. or multicenter study.pt. or clinical trial.pt. or exp Clinical Trials as topic/ or (clinical adj trial$).tw. or ((singl$ or doubl$ or treb$ or tripl$) adj (blind$3 or mask$3)).tw. or PLACEBOS/ or placebo$.tw. or randomly allocated.tw. or (allocated adj2 random$).tw.) not (case report.tw. or letter/ or historical article/)	1610881
12	Epidemiologic Studies/ or exp Case Control Studies/ or exp Cohort Studies/ or Case-control.tw. or (cohort adj (study or studies)).tw. or Cohort analy$.tw. or (Follow up adj (study or studies)).tw. or (observational adj (study or studies)).tw. or Longitudinal.tw. or Retrospective.tw. or Cross sectional.tw. or Cross-sectional studies/	3041720
13	11 OR 12	4234029
14	10 AND 13	7978
15	animals/ not (human/ and animals/)	4694218
16	14 NOT 15	7969

17	limit 16 to yr="2010 -Current"	**4716**

1-2. OVID-Embase 1974 to 2020 August 25

No.	Keywords	Results
1	exp Chronic Hepatitis C/	11108
2	chronic adj5 (hepatitis C or hepacivirus* or hcv).mp.	46284
3	1 OR 2	46284
4	exp Antivirus agent/	994602
5	exp Proteinase Inhibitor/	382195
6	exp Nucleic acid synthesis inhibitor/	413
7	(direct* acting anti?viral*) or DAA or ((protease or polymerase) adj3 inhibitor*).mp.	78454
8	(ledipasvir OR sofosbuvir OR elbasvir OR grazoprevir OR velpatasvir OR voxilaprevir OR glecaprevir OR pibrentasvir OR ombitasvir OR paritaprevir OR ritonavir OR dasabuvir OR simeprevir OR boceprevir OR telaprevir OR asunaprevir OR daclatasvir OR Harvoni OR Zepatier OR Epclusa OR Vosevi OR Mavyret OR Maviret OR “Holkira Pak”OR “Viekira Pak”OR Viekirax OR Exviera OR Technivie OR Galexos OR Sovaldi OR Victrelis OR Incivek OR Incivo OR Olysio OR Sunvepra OR Daklinza).mp.	43047
9	4 or 5 or 6 or 7 or 8	1305661
10	3 and 9	28135
11	(Clinical Trial/ OR Randomized Controlled Trial/ OR controlled clinical trial/ OR multicenter study/ OR Phase 3 clinical trial/ OR Phase 4 clinical trial/ OR exp RANDOMIZATION/ OR Single Blind Procedure/ OR Double Blind Procedure/ OR Crossover Procedure/ OR PLACEBO/ OR randomi?ed controlled trial$.tw. OR rct.tw. OR (random$ adj2 allocat$).tw. OR single blind$.tw. OR double blind$.tw. OR ((treble or triple) adj blind$).tw. OR placebo$.tw. OR Prospective Study/) not (Case Study/ OR case report.tw. OR abstract report/ or letter/ OR Conference proceeding.pt. OR Conference abstract.pt. OR Editorial.pt. OR Letter.pt. OR Note.pt.)	1749593
12	Clinical study/ OR Case control study OR Family study/ OR Longitudinal study/ OR Retrospective study OR (Prospective study/ not Randomized controlled trials/) OR Cohort analysis/ OR (Cohort adj (study or studies)).mp. OR (Case control adj (study or studies)).tw.OR (follow up adj (study or studies)).tw.OR (observational adj (study or studies)).tw.OR (epidemiologic$ adj (study or studies)).tw. OR (cross sectional adj (study or studies)).tw.	2815140
13	11 OR 12	4004982
14	10 AND 13	9051
15	animals/ not (human/ and animals/)	896577

16	14 NOT 15	9051
17	limit 16 to yr="2010 -Current"	**5985**

1-3. Cochrane Register of Controlled Trials (CENTRAL), 2020.8.25.

No.	Keywords	Results
1	[Hepatitis C, Chronic] explode all trees	1952
2	chronic near/5 (hepatitis C or hepacivirus* or hcv)	11679
3	#1 OR #2	11679
4	MeSH descriptor: [Antiviral Agents] explode all trees	8183
5	MeSH descriptor: [Protease Inhibitors] explode all trees	7032
6	MeSH descriptor: [Nucleic Acid Synthesis Inhibitors] explode all trees	1047
7	(direct* acting anti?viral*) or DAA or ((protease or polymerase) near/3 inhibitor*)	4293
8	(ledipasvir OR sofosbuvir OR elbasvir OR grazoprevir OR velpatasvir OR voxilaprevir OR glecaprevir OR pibrentasvir OR ombitasvir OR paritaprevir OR ritonavir OR dasabuvir OR simeprevir OR boceprevir OR telaprevir OR asunaprevir OR daclatasvir OR Harvoni OR Zepatier OR Epclusa OR Vosevi OR Mavyret OR Maviret OR 'Holkira Pak'OR 'Viekira Pak'OR Viekirax OR Exviera OR Technivie OR Galexos OR Sovaldi OR Victrelis OR Incivek OR Incivo OR Olysio OR Sunvepra OR Daklinza)	4560
9	#4 or #5 or #6 or #7 or #8	18905
10	#3 and #9	3266
11	#10 with Publication Year from 2010 to 2020, in Trials	**2063**

1-4. KMbase

No.	Keywords	Results
1	("hepatitis C" OR hepacivirus OR HCV) AND ("direct acting antiviral" OR DAA)	44
2	("hepatitis C" OR hepacivirus OR HCV) AND (ledipasvir OR sofosbuvir OR elbasvir OR grazoprevir OR velpatasvir OR voxilaprevir OR glecaprevir OR pibrentasvir OR ombitasvir OR paritaprevir OR ritonavir OR dasabuvir OR simeprevir OR boceprevir OR telaprevir OR asunaprevir OR daclatasvir OR Harvoni OR Zepatier OR Epclusa OR Vosevi OR Mavyret OR Maviret OR "Holkira Pak"OR "Viekira Pak"OR Viekirax OR Exviera	30

	OR Technivie OR Galexos OR Sovaldi OR Victrelis OR Incivek OR Incivo OR Olysio OR Sunvepra OR Daklinza)	
	**Overall results after excluding duplicates**	**61**
1	(“hepatitis C” OR hepacivirus OR HCV) AND “direct acting antiviral“	65
2	(“hepatitis C” OR hepacivirus OR HCV) AND DAA	41
3	(ledipasvir OR sofosbuvir OR elbasvir OR grazoprevir OR velpatasvir OR voxilaprevir OR glecaprevir OR pibrentasvir OR ombitasvir OR paritaprevir OR ritonavir OR dasabuvir OR simeprevir OR boceprevir OR telaprevir OR asunaprevir OR daclatasvir OR Harvoni OR Zepatier OR Epclusa OR Vosevi OR Mavyret OR Maviret OR “Holkira Pak”OR “Viekira Pak”OR Viekirax OR Exviera OR Technivie OR Galexos OR Sovaldi OR Victrelis OR Incivek OR Incivo OR Olysio OR Sunvepra OR Daklinza)	55
4	(([ALL=C 형 간염] OR [ALL=C 형간염]) AND ([ALL=항바이러스제] OR [ALL=항 바이러스제]))	14
	**Overall results after excluding duplicates**	120

1-5. RISS

No.	Keywords	Results
1	(“hepatitis C” OR hepacivirus OR HCV) AND “direct acting antiviral"	159
2	(“hepatitis C” OR hepacivirus OR HCV) AND DAA	189
3	(ledipasvir OR sofosbuvir OR elbasvir OR grazoprevir OR velpatasvir OR voxilaprevir OR glecaprevir OR pibrentasvir OR ombitasvir OR paritaprevir OR ritonavir OR dasabuvir OR simeprevir OR boceprevir OR telaprevir OR asunaprevir OR daclatasvir OR Harvoni OR Zepatier OR Epclusa OR Vosevi OR Mavyret OR Maviret OR “Holkira Pak”OR “Viekira Pak”OR Viekirax OR Exviera OR Technivie OR Galexos OR Sovaldi OR Victrelis OR Incivek OR Incivo OR Olysio OR Sunvepra OR Daklinza)	1
4	C 형 간염 AND 항 바이러스제	1
5	C 형간염 AND 항바이러스제	15
	**Overall results after excluding duplicates**	**240**

II. Supplementary Figures

(1) The risk of HBV reactivation by area

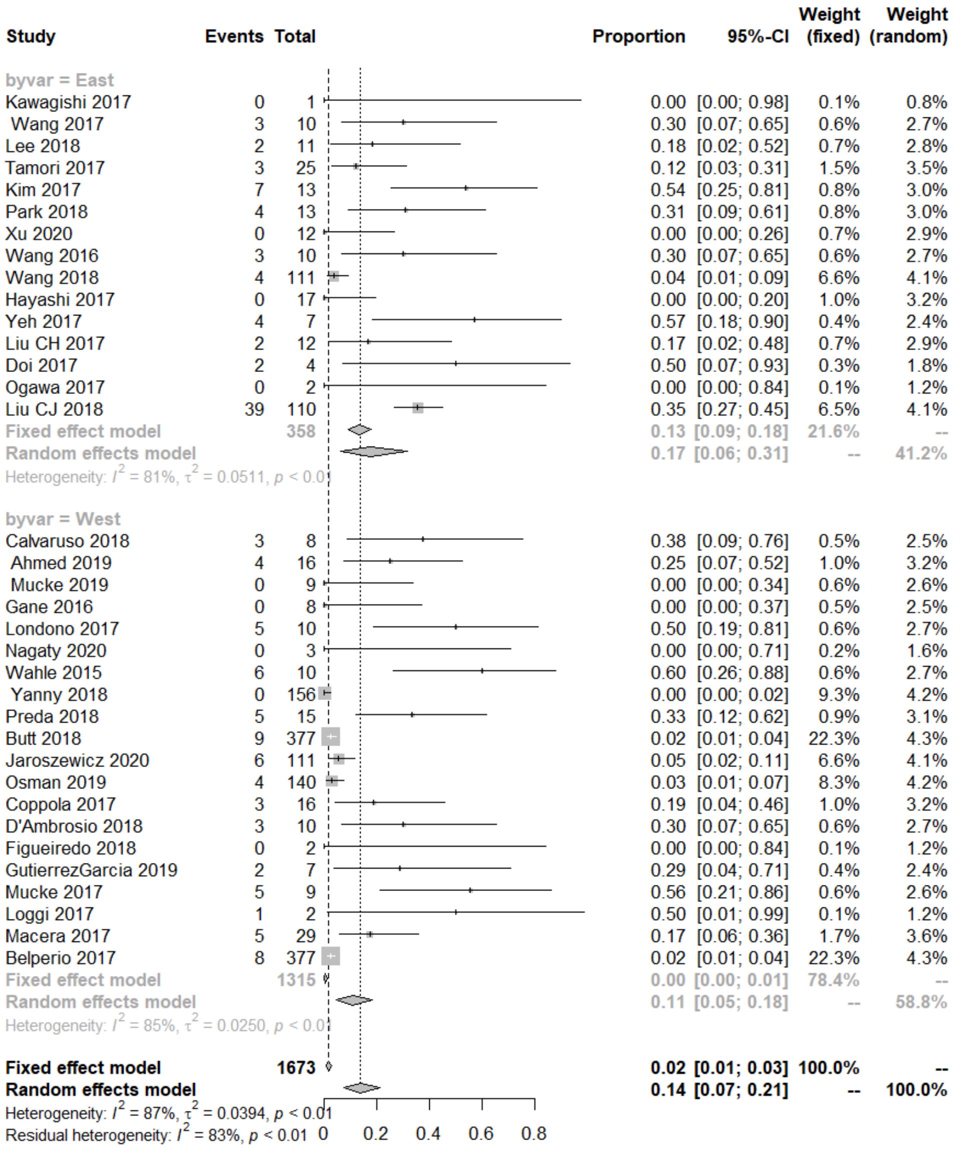

(2) Risk of bias

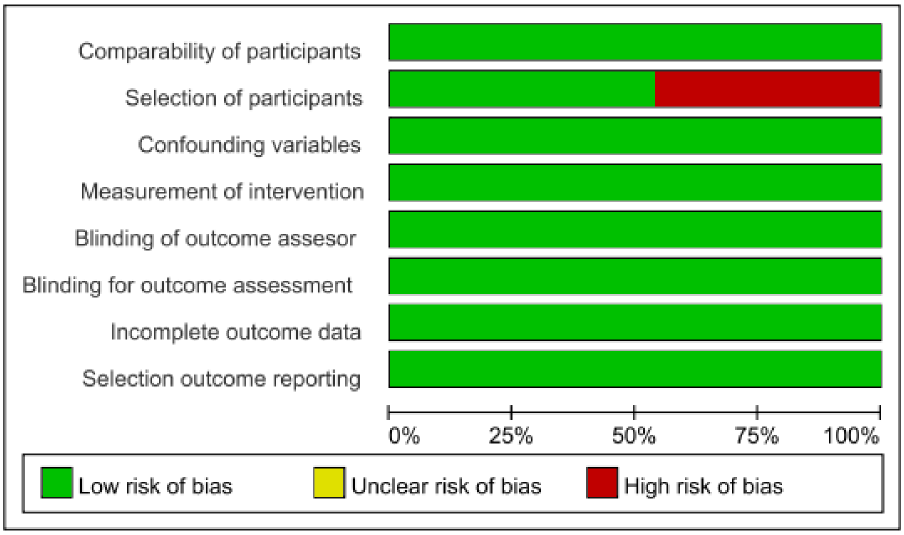

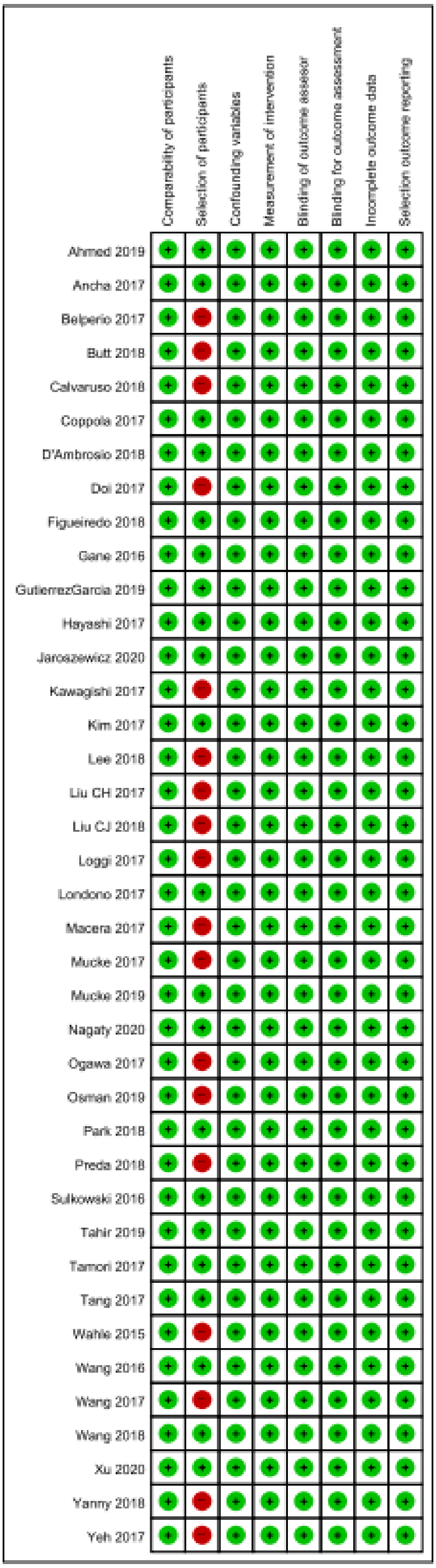
Comparability of participants
Selection of participants
Confounding variables
Measurement of intervention
Binding of outcome assessor
Binding for outcome assessment
Incomplete outcome data
Selection outcome reporting
Ahmed 2019
Ancha 2017
Belperio 2017
Butt 2018
Calvaruso 2018
Coppola 2017
D'Ambrosio 2018
Doi 2017
Figueiredo 2018
Gane 2016
GutierrezGarcia 2019
Hayashi 2017
Jaroszewicz 2020
Kawagishi 2017
Kim 2017
Lee 2018
Liu CH 2017
Liu CJ 2018
Loggi 2017
Londono 2017
Macera 2017
Mucke 2017
Mucke 2019
Nagaty 2020
Ogawa 2017
Osman 2019
Park 2018
Preda 2018
Sulkowski 2016
Tahir 2019
Tamori 2017
Tang 2017
Wahle 2015
Wang 2016
Wang 2017
Wang 2018
Xu 2020
Yanny 2018
Yeh 2017

(3) Funnel plot for analysis publication bias.

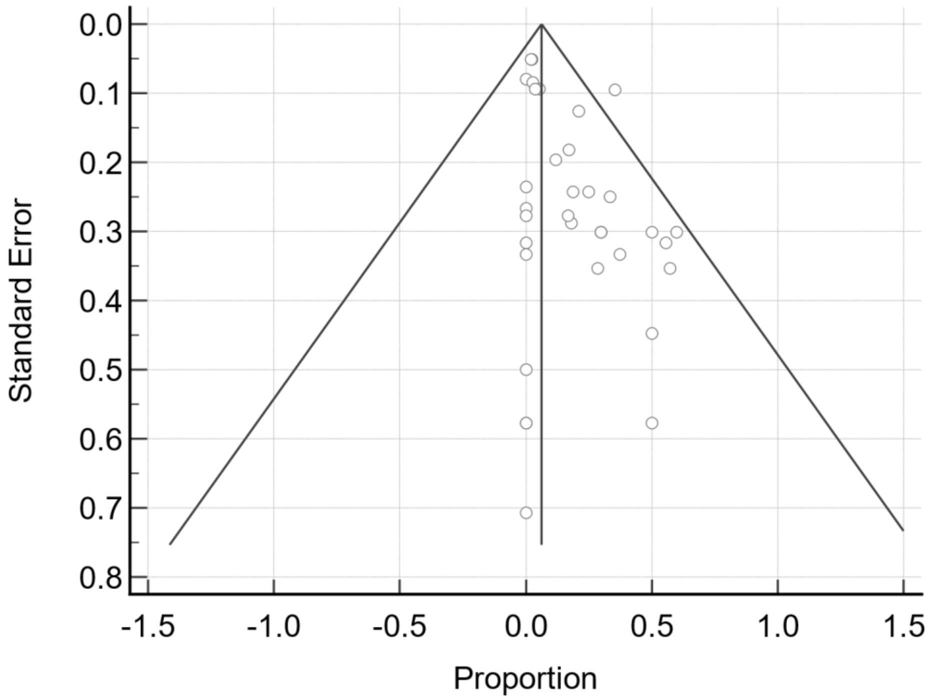

Egger's test: p value 0.0002
